# Nutritional management of glycogen storage disease type III: a case report and a critical appraisal of the literature

**DOI:** 10.3389/fnut.2023.1178348

**Published:** 2023-05-11

**Authors:** Elena Massimino, Anna Paola Amoroso, Roberta Lupoli, Alessandro Rossi, Brunella Capaldo

**Affiliations:** ^1^Department of Clinical Medicine and Surgery, University Federico II, Naples, Italy; ^2^Department of Molecular Medicine and Medical Biotechnology, University Federico II, Naples, Italy; ^3^Department of Translational Medicine, Section of Pediatrics, University Federico II, Naples, Italy

**Keywords:** glycogen storage disease, dietary intervention, high-fat diet, cardiomyopathy, myopathy, continuous glucose monitoring (CGM)

## Abstract

Glycogen storage disease Type III (GSD III) is an autosomal recessive disease due to the deficiency of the debranching enzyme, which has two main consequences: a reduced availability of glucose due to the incomplete degradation of glycogen, and the accumulation of abnormal glycogen in liver and cardiac/skeletal muscle. The role of dietary lipid manipulations in the nutritional management of GSD III is still debated. A literature overview shows that low-carbohydrate (CHO) / high-fat diets may be beneficial in reducing muscle damage. We present a 24-year GSD IIIa patient with severe myopathy and cardiomyopathy in whom a gradual shift from a high-CHO diet (61% total energy intake), low-fat (18%), high-protein (21%) to a low-CHO (32 %) high-fat (45%) / high-protein (23%) diet was performed. CHO was mainly represented by high-fiber, low glycemic index food, and fat consisted prevalently of mono and polyunsaturated fatty acids. After a 2-year follow-up, all biomarkers of muscle and heart damage markedly decreased (by 50–75%), glucose levels remained within the normal range and lipid profile was unchanged. At echocardiography, there was an improvement in geometry and left ventricular function. A low -CHO, high-fat, high-protein diet seems to be safe, sustainable and effective in reducing muscle damage without worsening cardiometabolic profile in GSDIIIa. This dietary approach could be started as early as possible in GSD III displaying skeletal/cardiac muscle disease in order to prevent/minimize organ damage.

## 1. Introduction

Glycogen storage disease Type III (GSD III) (#MIM 232400) is an autosomal recessive disease due to deficiency of the debranching enzyme (GDE) encoded by the AGL gene, located on chromosome 1p21. GDE allows the cleavage of glucose molecules from glycogen through two distinct catalytic activities: 1) the 1,4-α-D-glucan 4-α-D-glycosyl transferase, which transfers the terminal three glucose molecules to the parent chain and 2) the amylo-1,6-glucosidase component, which cleaves the alpha 1,6 bond to release free glucose. GDE deficiency has two main consequences: a reduced availability of energy /glucose due to the incomplete degradation of glycogen, and the accumulation in liver and cardiac/skeletal muscle of abnormal glycogen with short external ramifications (limit dextrin). Functionally, patients with GSD III have defective glycogenolysis while glycolysis and gluconeogenesis are preserved. There are two major clinical subtypes of GSD III: GSD IIIa (85% of cases)– involving liver and cardiac/skeletal muscle, and GSD IIIb, where only the liver is affected (1, 2). In childhood, the disease usually presents with ketotic hypoglycemia, hepatomegaly, growth retardation and elevated transaminase levels. Progressive skeletal myopathy, and often cardiomyopathy, appear in adulthood (3).

Traditionally, the dietary management of GSD III includes a high proportion of carbohydrates distributed throughout the day, together with uncooked cornstarch supplements to maintain euglycemia. Protein intake is also increased to provide substrates for neoglucogenesis (4). However, some criticisms have been raised regarding this dietary approach. First, a high-carbohydrate diet can induce reactive hyperinsulinemia with activation of glycogen synthesis and further accumulation of abnormal glycogen in the affected tissues, with worsening of organ damage; furthermore, carbohydrate overtreatment can lead to suppression of lipolysis, ketogenesis, and gluconeogenesis with reduced availability of alternative energy substrates. Based on these considerations, diets containing high amount of fat and/or protein have recently been proposed as a valid alternative to the traditional approach (5, 6). In particular, dietary lipid manipulations, including modified ketogenic diets with or without medium-chain triglycerides (MCT), have been shown to be associated with a reduction in liver enzymes, creatinkinase, and interventricular septum thickness, although with some variability between children and adults with GSD III (5).

Here, we present a patient with GSD IIIa, severe myopathy and heart failure in whom a gradual transition from a high CHO-diet to a high-fat high-protein diet was performed under careful monitoring of glucose and biochemical parameters. The main steps of dietary management and their impact on the course of organ damage are described.

## 2. Case presentation

The patient is a 24- year-old man with GSD IIIa, born to non-consanguineous parents. The diagnosis was made at age 9 months, based on 1–6 glycosidase deficiency in erythrocytes and confirmed by molecular analysis, which showed a homozygous IVS21 + 1A/G (c.2681 + 1G>A) mutation in the *AGL* gene.

Since the diagnosis, the patient was under follow-up at the Pediatric Unit of Federico II University Hospital in Naples. Over the years, the patient presented regular growth and development. Dietary regimen was based on high CHO intake, including administration of cornstarch. Starting with puberty, a progressive worsening of his muscle and cardiac biomarkers was observed. At age 16, due to the occurrence of asthenia and reduced exercise capacity, the patient underwent a cardiological assessment, which evidenced diffuse ventricular repolarization anomalies and signs of left ventricular hypertrophy. At the age of 22, the patient was referred to the outpatient clinic for rare metabolic diseases in adults of the Department of Clinical Medicine and Surgery of Federico II University in Naples for clinical management and follow-up. Upon admission, the patient underwent a careful assessment of his clinical status and complications, which was repeated during the dietary intervention according to the timeline reported in Table 1. Body weight was within the normal range, while fat-free mass (FFM) was slightly reduced. Resting energy expenditure (REE), evaluated through indirect calorimetry, was 22% higher than the predicted value calculated using the Harris Benedict equation. Glucose profile, monitored for 4 weeks by means of FreeStyle Libre 2 (Abbott), showed quite large glycemic fluctuations, ranging from 54 to 140 mg/dl (3–7.78 mmol/L), with 9% of time spent in hypoglycemia (< 70 mg/dl, < 3.89 mmol/L). Both plasma insulin and HOMA index were elevated, indicating a condition of insulin resistance. Lipid profile showed high levels of both cholesterol and triglycerides. Circulating markers of cardiac and skeletal muscle were markedly elevated. Echocardiography revealed increased LV mass and reduced global longitudinal strain (GLS), indicating myocardial injury. Cardiac MRI confirmed hypertrophic cardiomyopathy with preserved biventricular systolic function. AST and ALT were 5-fold higher than normal values and the liver ultrasound showed enlargement of the organ and diffuse hepatic steatosis. The patient's diet, evaluated through a 7-day food record, is reported in Table 2. Calorie intake was quite high (3132 Kcal/day) mainly due to an elevated intake of CHO (513 g/day, 61% of Total Energy Intake). The amount of cornstarch was 215 g/day (3.16 g/kg BW) divided into two administrations in daytime and one at nighttime, in addition, to 80g/day of maltodextrin – a high glycemic index (GI) polysaccharide sugar. Fiber intake was low (11 g/day), due to reduced consumption of fruit and vegetables. Protein intake was 2.4 g/kg BW (163 g/day, 21% TEI) of which 28% (45g/die) as a protein powder supplement (Protifar, Nutricia) divided into two daily doses. Total fat intake was rather low (18% TEI), with percentages of monounsaturated, polyunsaturated, and saturated fatty acids of 9, 2, and 5%, respectively.

**Table 1:** Timeline of clinical, metabolic and organ damage biomarkers before and during the nutritional intervention.

		**On admission (0 m)**	**1st step intervention (6 m)**	**After 1-yr COVID lock-down(18 m)**	**2nd step intervention (24 m)**	**Range values**
Antropometric	Weight (Kg)	68	68	70	70	
	BMI (Kg/m^2^)	23.5	23.5	24.2	24.2	20–25
	REE (Kcal/die)	2,073			1,862	
	REE (% predicted)	122%			110%	
	QR	0.9			0.9	
	FFM (Kg) (%)	50.2 (76)			51.4 (73)	80–90%
Metabolic	Glucose (mg/dl) (mmol/L)	96 (5.33)	80 (4.44)	86 (4.78)	94 (5.22)	70–110 (3.89–6.11)
	Time spent at IG 70–140 mg/dl (%)	91			96	
	Time spent at IG < 69 mg/dl (%)	9			4	
	Insulin (μU/ml)	41	15.2	10	12.8	3–25
	HOMA-index	9.7	3.0	2.1	3.0	
	Lactate (mol/L)	5.7	2.4	1.8	0.9	0.5–2.2
	Total Cholesterol (mg/dl) (mmol/L)	213 (5.5)	217 (5.61)	228 (5.89)	213 (5.5)	< 190 (4.91)
	HDL-Chol (mg/dl) (mmol/L)	35 (0.9)	37 (0.96)	37 (0.96)	39 (1.01)	>40 (1.03)
	LDL -Chol (mg/dl) (mmol/L)	153 (3.95)	166 (4.29)	176 (4.55)	151 (3.9)	< 115 (2.97)
	Triglyceride (mg/dl) (mmol/L)	205 (2.31)	206 (2.33)	172 (1.94)	197 (2.22)	< 150 (1.69)
Muscle/Heart	CK (U/L)	22,423	12,968	12,590	10,431	30–200
	LDH (U/L)	2,612	1,651	1,507	1,128	125–243
	CK MB (ng/ml)	>600	418	398	205	0–7.2
	Troponin I (pg/ml)	336	387	442	282	0–34
	Pro-BNP (pg/ml)	1,175	787	980	362	< 125
	LV Mass Index (g/m ^2.7^)	65			58	< 45 (F), 49 (M)
	Interventricular Septum (mm)	15			14	< 11
	Global Longitudinal Strain (%)	16,4			17,7	>20
Liver	AST (U/L)	292	226	229	220	0–34
	ALT (U/L)	216	181	239	255	0–55
	ALP (U/L)	191	174	165	150	40–150
	Liver longitudinal diameter (right/left lobe) (mm)	191/156			187/147	

IG, interstitial glucose; HOMA-Index (7).

**Table 2:** Daily dietary intake (7d-diary) on admission and during dietary intervention.

	**On admission (0 m)**	**After 1st step intervention (6m)**	**After 1-yr Covid lock-down (18m)**	**After 2nd step intervention (24 m)**
Energy (kcal) (KJ)	3,132 (13,114)	3,105 (13000)	2,870 (12016)	3,046 (12,752)
Carbohydrates (g) (%TEI)	513 (61)	422 (51)	396 (52)	260 (32)
Protein (g) (%TEI)	163 (21)	192 (25)	194 (27)	177 (23)
Lipid (g) (%TEI)	62 (18)	84 (24)	68 (21)	152 (45)
Monounsaturated (%TEI)	9	14	9	27
Polyunsaturated (%TEI)	2	4	4	6
Saturated (%TEI)	5	5	6	10
Corn starch (g)	215	100	100	80
Maltodextrin (g)	80	0	0	0
Fiber (g)	11	28	28	26

The patient was treated with oral L-Carnitine (2gx2 times /day) and Vitamin D (10,000 U /ml, 30 drops/wk).

## 3. Dietary intervention

We decided to implement a gradual shift from a diet with a high CHO content to one with a high fat content, under careful monitoring of glucose levels. Dietary changes were performed in 2 steps, each lasting 6 months, which were interrupted for 1 year, due to the COVID-19 pandemic, during which the patient partly adhered to the 1st dietary step.

Based on the results of the calorimetric assessment showing a high resting energy expenditure (REE), we prescribed an isocaloric diet to maintain body weight stable. During the 1st step, maltodextrins were progressively eliminated, and the cornstarch supplementation was reduced by more than 50%. In the 2nd step, the CHO content halved compared to initial levels (from 61 to 32% TEI) and cornstarch supplementation was gradually reduced to 1.1 g/kg bw/day under the guide of CGM data. Much attention was paid to CHO quality, preferring—among starchy sources—foods rich in fiber with a low glycemic index (wholemeal foods, legumes, vegetables). In parallel, lipid intake was progressively increased from 18% upon admission to 24% in the 1st step and to 45% in the 2nd step. Foods rich in monounsaturated and polyunsaturated fats (olive oil, almonds, nuts) were preferred, to achieve a percentage of saturated fatty acids below 10% of TEI, as recommended by international nutritional guidelines. Regarding protein intake, according to guidelines (4), we maintained a percentage of ≈25% TEI including a protein powder supplement. To reinforce the patient's adherence to dietary intervention, daily menus were provided (Appendix A).

At the end of each step, clinical, biochemical and imaging organ exams were performed to evaluate the efficacy of the dietary intervention.

## 4. Results

The patient well tolerated the dietary changes and showed a good adherence to prescriptions. He reported an improved feeling of wellbeing and greater exercise capacity (also evidenced by the SF36 questionnaire). The changes in clinical, metabolic and organ damage biomarkers during the nutritional intervention are presented in Table 1. Body weight remained substantially stable during the nutritional intervention (except for a 2-kg increase due to a more sedentary lifestyle during the lockdown due to COVID restrictions). Although REE decreased by ≈200 Kcal, it was still 10% higher than the predicted value). No significant changes were found in body composition. Since the 1^st^ step of dietary intervention, there was a remarkable reduction in insulin levels (from 41 to 15 μU/ml) and in insulin resistance index (from 9.7 to 3.0) resulting from the reduced CHO intake. It is noteworthy that despite the substantial reduction in CHO intake, no severe hypoglycemia occurred; instead, during the nutritional intervention both the number and the average duration of hypoglycemia (< 70 mg/dl, < 3.89 mmol/L) decreased (from 41 episodes/4 week on admission to 24 episodes/4 week after the 2nd step, and from 99 to 56 min, respectively). Notably, lipid profile remained unchanged despite the consistent increase in fat intake. Since the 1^st^ step intervention, both muscle and cardiac enzymes markedly decreased, with a 50% reduction in CK (from 22,423 to 10,431 U/L) and LDH (from 2,612 to 1,128 U/L) and a 70% reduction in Pro-BNP (from 1,175 to 362 pg/ml). Cardiac ultrasound evidenced an improvement in geometry and LV function as evidenced by a significant reduction in LV mass and an increase in global longitudinal strain (GLS). Liver enzymes remained substantially stable and a trend toward a reduction in liver size was found at ultrasound examination.

## 5. Discussion

By chronologically reviewing the literature on the dietary management of patients with GSD III (Table 3), it is evident that over the years several attempts have been made to define the best nutritional approach capable of maintaining euglycemia and, in the meantime, preventing/limiting the long-term complications of the disease involving heart, muscle and liver. Unfortunately, the analysis of the available literature does not lead to firm conclusions due to a large variation in patients' ages (from 2 months to 47 years) (8, 9), duration of follow-up (from 4 months to 5 years), types of nutritional intervention—which are often not sufficiently detailed in terms of bromatological composition and, finally, the clinical outcomes (heart, muscle, liver).

**Table 3:** Chronological overview of clinical case reports of patients with GSD III treated with dietary manipulation.

**Study**	**Patients**	**Diagnosis**	**Diet description**					**Follow-up (months)**	**Main clinical benefits**
			**Kcal**	**CHO**	**Prot**	**Fat**	**Supplements**		
Slonim (10)	♂ 7-yr	Debrancher deficiency (Liver and Muscle biopsy)	1,600	50%	25%	25%	Sustacal^*^	20	- Improved muscle strength and growth.
Kiechl (9)	♀ 47-yr	Debrancher deficiency (Muscle biopsy)	nr	nr	30–35%	nr		4	- CK ↓ - normalization of muscle strength and spirometry
Dagli (11)	♂ 22-yr	Retention of limit dextrin in cultured fibroblasts	nr	nr	30%	nr	Cornstarch: 1,36g/Kg	12	- CK ↓ - Decreased Left ventricular mass index
Valayannopoulos (8)	♂ 2 months	Homozygosity for the mutation: c.2157+IG>T	nr	20%	15%	65%	3OHB at the dose of 400 to 800 mg/Kg/d	24	- Decreased Interventricular wall thickness
Sentner (12)	♀ 32-yr	Homozygosity for the mutation: c.753_756delCAGA	900^§^	61%	37%	2%		4	-CK ↓ - Decreased Interventricular Septum (IVS)
			1,370	nr	4,3%	nr	Cornstarch: 2 doses	32	
Mayorandan (15)	1) ♂ 9-yr	Homozygosity for the mutation: c.4256dupC	nr	0,4g/Kg	7g/Kg	8g/Kg		32	-CK ↓ -ProBNP ↓ - Decreased Ventricular Septum Thickness
	2) ♂ 11-yr	Homozygosity for the mutation: c.753_756del	nr	0,5g/Kg	6g/Kg	5g/Kg		26	Dietary noncompliance.
Brambilla (19)	1) ♀ 7-yr	Homozygosity for the mutation: p.G1087R	1,120	15%	26%	5,9%	Protein powders	12	- CK ↓ - Decreased IVS
	2) ♂ 5-yr		1,050	15%	25%	6,0%			- CK ↓ - Decreased IVS
Francini-Pesenti (16)	♂ 34-yr	nr	nr	20g /day	nr	nr	Cornstarch in case of hypoglycemia	12	- CK ↓ - Increased Steps/24h - Improved ejection fraction
Fisher (18)	1) ♂ 37-yr	Homozygosity for the mutation: (c.753_756delCAGA; p.ASP251Glufs^*^23)	nr	5-20%	9-23%	70-78%	MCT: 11-23% of total daily fat	60	- CK ↓ -Stabilization of blood glucose
	2) ♂ 40-yr								
Marusic (21)	♀ 15-yr	Homozygosity for the mutation: c.3980G>A	nr	2%	11%	87%		48	- Improved Hepatic, Muscle and Cardiac markers - Decreased Left Ventricular Mass Index and septal Wall
Olgac (17)	1) ♂4,5-yr 2) ♂11-yr 3) ♂11-yr 4) ♂9-yr 5) ♀ 31-yr 6) ♀ 3-yr	Nr	nr	10%	20%	70%	Vitamins and minerals	Range: 3–7	- CK ↓
Kumru Akin (20)	♂ 9-yr	Homozygosity for the mutation: c.1783C > T	1,400	30%	2,0%	50%	Protifar^**^ (1g/Kg) and Glycosade^®^^ç^ (2g/Kg).	18	- CK ↓ - Improved left ventricular outflow tract - Decreased interventricular septum

^*^Sustacal contains 24% protein, 55% carb. and 21% fat.

^**^Protifar contains 20% casein and 80% whey.

^Ç^Cornstarch (Vitaflo International Ltd. Liverpool, UK).

The hallmark of all these dietary programs is a reduction in CHO intake associated with an increase in protein and, more recently, in fat intake. The rationale being to avoid excessive glycemic elevation with consequent reactive hyperinsulinemia and tissue deposition of abnormal glycogen and, in the meanwhile, to provide alternative fuel for body needs.

The clinical benefits of a high protein diet were first demonstrated in a case report showing an improvement in strength and muscle mass in a 7-year-old patient receiving a protein intake of 25% of TEI (10). Another clinical case reported a partial remission of subacute respiratory failure in a 47-year-old GSD III patient receiving a 30–35% protein diet (9). Subsequently, the benefits of a high protein diet (30–37% TEI) were described in a patient with hypertrophic cardiomyopathy at pre-transplant stage (11, 12). More recently, a high fat diet has gained much attention following the evidence from cardiovascular research that ketone bodies are an efficient metabolic substrate for the failing heart since they require less oxygen per molecule of ATP generated (13). In addition, the administration of D,L-3-hydroxybutyrate was found to be beneficial in patients with cardiomyopathy secondary to defects of fatty acid oxidation (14). Finally, Valayannopoulus et al. published the successful treatment of a 2-month-old infant with severe cardiomyopathy, by means of ketone bodies supplementation (D, L-3-hydroxybutyrate), ketogenic and high protein diet (8). Overall, these data lent support to the hypothesis that a high-fat diet may help control myocardiopathy in patients with GSD III. Moving from this belief, different approaches based on variable fat intakes have been tested in patients with GSD III: the Modified Atkins Diet (MAD) (15–17), a MAD with MCT oil supplements (18), and high fat / high-protein diet (19, 20). The results show the efficacy of these approaches in reducing muscle enzymes and improving cardiomyopathy, although some concern related to patient compliance and long- term effects of these diets on bone health and lipid profile remain to be addressed. Indeed, the effect of nutritional modifications on traditional outcome parameters need to be carefully balanced against psychosocial wellbeing and quality of life in patients with hepatic GSDs (22).

The need for new therapies that can improve the quality of life of patients with GSD has prompted research toward innovative therapies such as the use of gene therapy or therapy using messenger RNA (mRNA). In principle, GSD patients would be no longer dependent on a strict dietary regimen. Early phase clinical studies are already available for GSD Ia and II and the approach appears promising also for other types of GSDs (23).

Our case report describes a patient with GSD IIIa with myopathy and severe hypertrophic cardiomyopathy in whom a gradual shift from a CHO-rich diet (61% TEI) to a high- fat (45% TEI) / high-protein (23% TEI) diet—performed under careful monitoring of glycemic and biochemical parameters—produced relevant clinical benefits and reduced organ damage. Indeed, both muscle and cardiac enzymes decreased by 50–70% and an improvement in geometry and LV function was found at echocardiography.

It is interesting to note that even a small reduction in CHO intake (from 61 to 51% TEI) together with the use of wholemeal products with a low glycemic index, were able to reduce the biochemical markers of muscle and heart damage. The unusually high CK values of our patient, compared to other cases reported, dropped from 22,423 to 12,968 U/L after only 6 months of dietary intervention. This improvement persisted throughout the COVID-19 pandemic, during which the patient kept his diet constant for a year. When CHO intake was reduced further (from 51 to 32% TEI) the biochemical parameters continued to improve, providing additional benefits. It is also interesting to note that, although lipid intake was increased from 18 to 45% of TEI, the lipid profile did not worsen, probably due to consumption of foods rich in monounsaturated and polyunsaturated fatty acids. These findings highlight the concept that beyond restricting CHO and increasing fat intake, the quality of foods is of paramount importance. In fact, low glycemic index foods and whole grains help keep glucose levels more stable, thus limiting the risk of hypoglycemia, while increasing mono- and polyunsaturated fat consumption prevents atherogenic modifications of the lipid profile– a very important finding considering that the nutritional therapy is life-long. Noteworthy, the number and duration of hypoglycemic episodes decreased during the dietary intervention despite the reduction in CHO intake. Particularly, an increase in Time In Range (TIR: 70–140 mg/dl, 3.89–7.78 mmol/L) was observed. Although optimal TIR for patients with GSD III has not been defined yet, a glycemic target for TIR has been recently proposed for adult patients with GSD Ia. Notably, the TIR observed in the present case was higher than that reported in GSD Ia patients (i.e., 96 vs. 83%), highlighting the lower contribution of liver involvement to disease phenotype in adult GSD III as compared to GSD I. CGM data from the present study support the opportunity to decrease CHO intake in older GSD III with no risk of precipitating hypoglycemia (24).

The finding of an REE value 22% higher than the predicted one observed in our patient deserves a comment. We first ruled out possible thyroid dysfunction and/or subclinical inflammatory diseases, which are known to increase energy expenditure. A possible explanation for the high REE value is that it may be related to an increased cell mass and, thus, to be an expression of organomegaly. Elevated REE values have been documented also in patients with GSD Ia (25, 26) and in patients with lysosomal storage disorders (27, 28), which are characterized by intra-organ accumulation of anomalous molecules. The slight reduction in REE (−200 kcal) during the nutritional intervention in conjunction with an albeit modest reduction in organ volumes supports this interpretation.

In conclusion, low-CHO high fat / high- protein diet showed a good efficacy in reducing organ damage and improving quality of life in our patient with GSD IIIa and severe cardiomyopathy. In addition to the low CHO intake, it is important to focus on macronutrient quality, favoring low-glycemic index food and unsaturated fats in order to preserve cardiometabolic health in the long-term. As a low CHO diet does not appear to increase the risk of hypoglycemia and is safe, this dietary approach could be started as early as possible in GSD III displaying skeletal/cardiac muscle disease in order to prevent/minimize organ damage.

## Data availability statement

The original contributions presented in the study are included in the article/Supplementary material, further inquiries can be directed to the corresponding author.

## Ethics statement

Ethical review and approval was not required for the study on human participants in accordance with the local legislation and institutional requirements. The patients/participants provided their written informed consent to participate in this study. Written informed consent was obtained from the participant/patient(s) for the publication of this case report.

## Author contributions

EM and APA collected the clinical data, carried out the literature search, and wrote a first draft. AR and RL reviewed the manuscript. BC took care of the patient, reviewed the manuscript, and provided relevant intellectual contribution. All authors have read and agreed to the published version of the manuscript.
