# Supplementary material for: Nutritional management of glycogen storage disease type III: a case report and a critical appraisal of the literature
Source: Front Nutr. 2023 May 11;10:1178348. doi: 10.3389/fnut.2023.1178348 (PMC10213733; doi:10.3389/fnut.2023.1178348)
Supplement: Supplementary file 1 [file Table_1.docx]

Supplementary Material

Appendix A

**Nutritional management of Glycogen Storage Disease Type III:**

**a case report and a critical appraisal of the literature**

**Elena Massimino^1^**, **Anna Paola Amoroso^1^**, **Roberta Lupoli^2^**, **Alessandro Rossi^3^**, **and Brunella Capaldo ^1*^**

**Correspondence:** Brunella Capaldo: brunellacapaldo@gmail.com

| 1^st^ dietary intervention sample | | |
| --- | --- | --- |
| Breakfast 7:30 | | low-fat milk g 180 |
|  |  | biscuits g 55 |
|  |  | Protifar g 25 |
| Morning snack 10:00 | | wholemeal sandwich g 70 |
|  |  | canned fish g 25 or ham g 20 |
| Lunch 13:00 | | Pasta g 80, tomato sauce g 50 |
|  |  | meat g 180 or fish g 180 |
|  |  | vegetables g 100 |
|  |  | fruits g 100 |
|  |  | extra virgin olive oil g 30 |
| Afternoon snack 17:00 | wholemeal sandwich 120 g | |
|  | canned fish g 50 or ham g 40 | |
| Dinner 20:00 | Bread g 100 | |
|  | meat g 180 or fish g 180 | |
|  | vegetables g 100 | |
|  | fruits g 100 | |
|  | extra virgin olive oil g 30 | |
| Evening snack 1:15 | low-fat milk 180 g | |
|  | wholemeal biscuits g 70 | |
|  | Protifar g 20 | |
|  | Corn starch 100 g | |

| 2^nd^ dietary intervention sample | |
| --- | --- |
| Breakfast 7:30 | low-fat milk g 180 |
|  | biscuits g 3 |
|  | Protifar g 20 |
| Morning snack 10:00 | 1 slice of wholemeal sandwich bread g 20 |
|  | canned fish g 25 or ham g 20 |
|  | extra virgin olive oil g 10 |
|  | walnuts g 15 |
| Lunch 13:00 | Pasta g 70, tomato sauce g 50 |
|  | meat g 180 or fish g 180 |
|  | vegetables g 100 |
|  | fruits g 100 |
|  | extra virgin olive oil g 30 |
|  | hazelnuts g 6 |
| Afternoon snack 17:00 | 1 slice of wholemeal sandwich bread g 20 |
|  | canned fish g 25 or ham g 20 |
|  | extra virgin olive oil g 10 |
|  | almonds g 7 |
| Dinner 20:00 | Bread g 90 |
|  | meat g 180 or fish g 180 |
|  | vegetables g 100 |
|  | fruits g 100 |
|  | extra virgin olive oil g 30 |
|  | hazelnuts g 6 |
| Evening snack 00:30 | 1 slice of wholemeal sandwich bread g 20 |
|  | canned fish g 25 or ham g 20 |
|  | extra virgin olive oil g 10 |
|  | almonds g 7 |
| Evening snack 1:15 | Low-fat milk g 180 |
|  | Protifar g 15 |
|  | Corn starch g 80 |

.
